# A Biologically-Inspired Model to Predict Perceived Visual Speed as a Function of the Stimulated Portion of the Visual Field

**DOI:** 10.3389/fncir.2019.00068

**Published:** 2019-10-30

**Authors:** Fabio Solari, Martina Caramenti, Manuela Chessa, Paolo Pretto, Heinrich H. Bülthoff, Jean-Pierre Bresciani

**Affiliations:** ^1^Department of Informatics, Bioengineering, Robotics, and Systems Engineering, University of Genova, Genoa, Italy; ^2^Department of Neuroscience and Movement Science, University of Fribourg, Fribourg, Switzerland; ^3^Istituto di Bioimmagini e Fisiologia Molecolare, Consiglio Nazionale delle Ricerche, Segrate, Italy; ^4^Virtual Vehicle Research Center, Graz, Austria; ^5^Department of Cognitive and Computational Psychophysics, Max Planck Institute for Biological Cybernetics, Tubingen, Germany; ^6^University Grenoble Alpes, LPNC, Grenoble, France

**Keywords:** vision, optical flow, motion perception, field of view, computational model, MST area

## Abstract

Spatial orientation relies on a representation of the position and orientation of the body relative to the surrounding environment. When navigating in the environment, this representation must be constantly updated taking into account the direction, speed, and amplitude of body motion. Visual information plays an important role in this updating process, notably via optical flow. Here, we systematically investigated how the size and the simulated portion of the field of view (FoV) affect perceived visual speed of human observers. We propose a computational model to account for the patterns of human data. This model is composed of hierarchical cells' layers that model the neural processing stages of the dorsal visual pathway. Specifically, we consider that the activity of the MT area is processed by populations of modeled MST cells that are sensitive to the differential components of the optical flow, thus producing selectivity for specific patterns of optical flow. Our results indicate that the proposed computational model is able to describe the experimental evidence and it could be used to predict expected biases of speed perception for conditions in which only some portions of the visual field are visible.

## 1. Introduction

Spatial orientation is a cognitive function based on the ability to understand, manipulate, visually interpret, and reorganize spatial relationships (Tartre, 1990). It relies on a representation of the position and orientation of the body relative to the surrounding environment and requires a mental readjustment of one's perspective to become consistent with the representation of a visually presented object (McGee, 1979; Tartre, 1990). When navigating in the environment, this representation must be constantly updated taking into account different aspects of body motion, such as its direction, speed and amplitude. Spatial navigation is a complex process that requires the integration of sensory information provided by different sensory channels such as vision, proprioception and the vestibular system. Visual information plays a particularly important role in this updating process, notably via the integration of optical flow information. Optical flow may be defined as the array of optical velocities that surround the moving subject (Kirschen et al., 2000), and it refers to the visual apparent motion between the body and the environment. Its characteristics are related not only to the speed and direction of motion, but also to the properties of the environment, such as for instance texture gradients. Optical flow information is particularly important for human locomotion, where it is integrated by the central nervous system, along with visual, vestibular, motor, kinesthetic and auditory signals, to give rise to motion perception (Mergner and Rosemeier, 1998). The alteration or manipulation of one of these signals may lead to an altered perception. In fact, studies that investigated how visual and non-visual/kinesthetic signals are integrated for speed perception with walking (Thurrell et al., 1998; Banton et al., 2005; Durgin et al., 2005; Kassler et al., 2010; Powell et al., 2011) and running participants (Caramenti et al., 2018, 2019) consistently reported an altered perception of visual speed.

One factor that has repeatedly been shown to affect perceived visual speed is the size of the field of view (FoV). Specifically, several studies demonstrated that peripheral vision is fundamental for motion perception. Indeed, the size of the FoV affects navigation abilities (Alfano and Michel, 1990; Cornelissen and van den Dobbelsteen, 1999; Turano et al., 2005), postural control (Dickinson and Leonard, 1967; Amblard and Carblanc, 1980; Stoffregen, 1986; Wade and Jones, 1997), speed perception (Osaka, 1988; Pretto et al., 2009) as well as vection, i.e., the self-motion perception induced by moving visual stimuli (Brandt et al., 1973; Berthoz et al., 1975; Held et al., 1975). Regarding speed perception in particular, smaller FoVs have been shown to induce a larger underestimation of visual speed, and this with walking (Thurrell et al., 1998; Thurrell and Pelah, 2002; Banton et al., 2005; Nilsson et al., 2014), cycling (Van Veen et al., 1998) and sitting still individuals (Pretto et al., 2009). Such reduction of the FoV can occur not only with simulated optical flows, due to the restrictions of the visualization device (e.g., screen, head-mounted displays), but also in medical conditions such as scotoma, in which there is a localized defect (i.e., blind spot) in the visual field that is surrounded by an area of normal vision.

Here we present a study in which we systematically investigated how the size and the simulated portion of the FoV affect perceived visual speed with human observers. In contrast to previous studies that only focused on the effect of the size of the FoV on visual speed perception, we also investigated the perceptual differences associated to the visible portion of the FoV. We propose a biologically-inspired computational model to account for the observed perceptual patterns. Different computational models have been suggested to qualitatively explain human visual speed perception. These models commonly assume that the perception of visual motion is optimal either within a deterministic framework with a regularization constraint that causes the solution to bias toward zero motion (Yuille and Grzywacz, 1988; Stocker, 2001), or within a probabilistic framework of Bayesian estimation with a prior that favors slow velocities (Simoncelli, 1993; Weiss et al., 2002). Stocker and Simoncelli (2005) presented a refined probabilistic model that can account for trial-to-trial variabilities that are typically observed in psychophysical speed perception problems. It is worth noting that these models take into account neural mechanisms that can be related to V1 and MT neural areas. However, to model the speed perception of motion patterns that are common in self-motion, we have to consider also the dorsal MST area, which encodes visual cues to self-motion (e.g., expansion and contraction) (Duffy and Wurtz, 1991; Pitzalis et al., 2013; Cottereau et al., 2017). Thus, we propose a computational model that takes into account the dorsal neural pathway (Goodale and Westwood, 2004), specifically V1, MT and MST areas, and the spatial non-linearity of log-polar mapping (Schwartz, 1977) in order to mimic the patterns of the perceived visual speed of human observers.

## 2. Materials and Methods

### 2.1. Perceived Visual Speed of Human Observers

#### 2.1.1. Participants

Eight participants aged 19–31 (mean=24.5 ± 3.82) participated in the experiment. All participants had normal or corrected-to-normal vision, and they were naive as to the purpose of the research. Written informed consent was obtained from all participants before their inclusion in the study. The experiment was performed in accordance with the ethical standards laid down in the 1964 Declaration of Helsinki, and approved by the ethical committee of the University of Tuebingen. The participants were paid, and they had the option to withdraw from the study at any time without penalty and without having to give a reason.

#### 2.1.2. Experimental Setup

**Figure 1:**
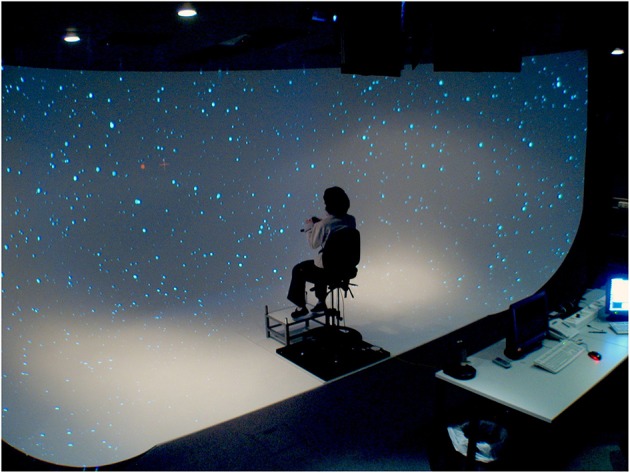
Panoramic screen, 230 × 125° of field of view, including floor.

The participants seated at the center of a panoramic screen (quarter of sphere) surrounding them in order to cover almost their entire visual field (see Figure 1). Specifically, the screen was cylindrical with a curved extension onto the floor, which provided a projection surface of 230° horizontally and 125° vertically. The screen surface was entirely covered by four LCD projectors with a resolution of 1,400 × 1,050 pixels each, and OpenWARP technology (Eyevis, Reutlingen, Germany) was used to blend overlapping regions. The height of the seat was adjusted so that eye height was 1.7 m for each participant. The geometry of the visual scene was adjusted for this eye height and a distance of 3 m of the vertical portion of the screen, i.e., the portion that is perpendicular to the floor and to the line of sight when looking straight ahead. These adjustments were made to avoid geometrical distortions induced by the curved display. The visual scene was generated using the Virtools software (Dassault Systemes SE) version 4.1.

#### 2.1.3. Visual Stimuli

The visual stimuli consisted of random patterns of either:

1. White dots generated as point sprites, which subtended a visual angle of a fifth of a degree, i.e., 12 arcminutes. The visual angle subtended by the dots (i.e., retinal size) did not change with distance from the viewer (Dots condition).
2. White 3D spheres, the “physical” size of which was 10 cm. The visual angle subtended by the spheres depended on the distance from the viewer (3D spheres condition).

Dots and 3D spheres were randomly located within a large virtual cube. The movement of a virtual camera through the dots induced a radial visual flow corresponding to a self-translation along the antero-posterior axis of the subject. Note that the eccentricity of the dots/spheres with respect to the fixation point varied from 0 to 115°, i.e., the half of the horizontal visual field of the panoramic screen used for the experiment. Accordingly, the speed profile of each dot/sphere varied from 0 m per second at a 0° eccentricity to the speed of the generated flow at a 180° eccentricity. The near clipping plane of the camera was set at 0.5 m, and the far clipping plane at 500 m. In the Dots condition, because the retinal size of the dots was distance-independent, the optical flow did not provide visual expansion cues. On the other hand, the 3D spheres provided visual expansion cues because the retinal size of the spheres increased as they moved closer to the viewer. A central fixation cross subtending 1.5° of visual angle and located in front of the participant at eye level was visible for the whole duration of the trials. The fixation cross corresponded to the focus of expansion of the optical flow.

#### 2.1.4. FoV Conditions

**Figure 2:**
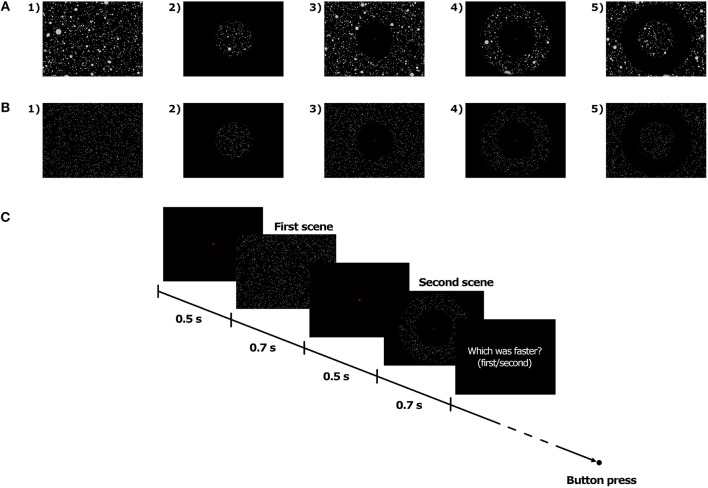
Illustration of the FoV condition with 3D spheres **(A)** and dots **(B)**, and time course of trials **(C)**.

Soft-edge disc-shaped transparent masks were implemented in the visual scene in order to manipulate the extent of the visible area on the screen. In the Full field of view (FoV) condition, the optical flow, whether consisting of Dots or 3D spheres, was visible on the whole screen (see Figures 2A1,B1). In the other FoV conditions, the masks were combined in order to generate four different types of optical flow. In the 10, 40, and 70C FoV conditions, only the central 10, 40, and 70° of the visual scene, respectively, displayed the optical flow (see Figures 2A2,B2). The 10, 40, and 70P FoV conditions corresponded to the exact opposite, and the central 10, 40, and 70° of the visual scene, respectively, were masked, so that the optical flow was visible only in the periphery of the mask (see Figures 2A3,B3). In the 10P40C, 10P70C, and 40P70C FoV conditions, the central and peripheral part of the visual scene were masked, and the optical flow was visible only in a ring-shaped of 10–to–40°, 10–to–70°, and 40–to–70°, respectively (see Figures 2A4,B4). Finally, in the 10C40P, 10C70P, and 40C70P FoV conditions, both the central and the peripheral part of the visual scene were visible, while a ring-shaped area of 10–to–40°, 10–to–70°, and 40–to–70°, respectively, was masked, so that no optical flow was displayed in the masked area (see Figures 2A5,B5). In all FoV conditions, the disc-shaped masks and rings were centered on the fixation cross, i.e., on the focus of expansion of the optical flow.

#### 2.1.5. Procedure

The stimuli were presented using a two-interval forced-choice (2-IFC) method. For each trial, two stimuli, namely a standard and a comparison stimulus, were successively presented to the participant. Both stimuli were moving at constant speed. At the end of the trial, the participant had to indicate in which interval (i.e., first or second) the stimulus was moving faster. For all FoV conditions, the standard stimulus was with Full FoV and it always moved at 5 m/s, i.e., 18 km/h. On the other hand, the speed of the comparison stimulus varied from trial to trial. Specifically, the speed of the comparison stimulus was determined for each trial by a Bayesian adaptive staircase (Kontsevich and Tyler, 1999), which took into account the speed of the previous visual stimuli as well as the corresponding responses of the participants. This method is based on an algorithm that optimizes the information gained with the previous trials. The Fov of the comparison stimulus was defined by the FoV condition (see FoV conditions).

At the beginning of each interval, the fixation cross appeared on a dark background. Participants were instructed to gaze at the cross and maintain the fixation until the end of the trial. 500 ms later, the first moving stimulus was presented for 700 ms, which included a 100 ms fade-in phase at the beginning and a 100 ms fade-out phase at the end. The second moving stimulus was presented 500 ms after the end of the first stimulus and had the same temporal structure as the first one. The fixation cross disappeared at the end of the second stimulus. The participant could then give its response by pressing on one of the two buttons of a joystick (i.e., first stimulus vs. second stimulus was faster). The time course of trials is presented in Figure 2C.

For each combination of visual stimulus (i.e., Dots vs. 3D spheres) and FoV condition (13 in total, see above), the adaptive staircase of the 2IFC method consisted of 80 trials. In total, the experiment consisted of 26 staircases of 80 trials each. The 26 staircases were split over two sessions that were run on two different days. The 13 staircases with Dots were all run on 1 day (Dots session), and the 13 staircases with 3D spheres (3D spheres session) were run on another day. Half of the subjects started with the Dots session, and the other half started with the 3D spheres session. For each type of visual stimulus/session, the 1040 trials (i.e., 13 staircases × 80 trials) were randomly interleaved, and presented in 8 blocks of 130 trials each. Each block lasted about 10 min with a 5 to 10 min break in between two consecutive blocks, so that in total, a session lasted about 2 h. During the breaks, the lights of the experimental room were switched on and subjects could walk and relax.

#### 2.1.6. Statistical Analyses

For each condition, the perceived speed was measured as the Point of Subjective Equality (PSE), i.e., the speed at which the comparison stimulus was perceived to move as fast as the standard stimulus. Note that when the PSE is higher than the actual speed of the standard stimulus, it indicates that the comparison stimulus was perceived as moving slower than the standard stimulus. Conversely, when the PSE is lower than the actual speed of the standard stimulus, it indicates that the comparison stimulus was perceived as moving faster than the standard stimulus. Both for the Dots condition (i.e., optical flow only) and for the 3D spheres condition (i.e., optical flow + expansion cues), mean PSEs were compared using either a one-way repeated measures analysis of variance (ANOVA) when data was parametric, or a Friedman rank sum test when data was not parametric. *Post-hoc* paired-comparisons were then performed using either Bonferroni correction for multiple comparisons (parametric data) or Friedman multiple comparisons test (non-parametric data). Additionally, a linear mixed model was used to directly compare the dots condition with the spheres condition. For all tests (except for the linear mixed model), in order to determine whether to use parametric (i.e., ANOVA) or non-parametric (i.e., Friedman test) methods of mean comparison, the normality of the residuals was assessed using the Shapiro-Wilk test, and *p*-values were Huynh-Feldt-corrected when the sphericity assumption was violated (as assessed with the Mauchly's test). All statistical tests were performed using the R statistical software.

### 2.2. Computational Model of Motion Processing

The proposed model, based on bio-inspired paradigms, describes a neural architecture that mimics the psychophysical outcomes of the previously described experiment that assess the influence of the size of the field of view on motion perception.

The neural architecture is composed of hierarchical cell layers that model the processing stages of the dorsal visual pathway (Goodale and Westwood, 2004; Orban, 2008). The activity of the MT area can be modeled by a V1-MT feed forward architecture. In particular, we can model V1 cells by using the motion energy model, based on spatio-temporal filtering, and MT pattern cells by pooling V1 cell responses (Adelson and Bergen, 1985; Simoncelli and Heeger, 1998; Solari et al., 2015; Chessa et al., 2016b). Then, the neural activity of the MT area is processed by populations of MST cells that have selectivity for specific patterns of optical flow: in particular, they can be sensitive to the differential components of the optical flow (Grossberg et al., 1999; Beardsley and Vaina, 2001; Chessa et al., 2013). The selectivity of the MST cells can be related to the relative motion between an observer and the scene, in particular to the speed of forward translation during self-motion through the environment (Chessa et al., 2013, 2016a).

**Figure 3:**
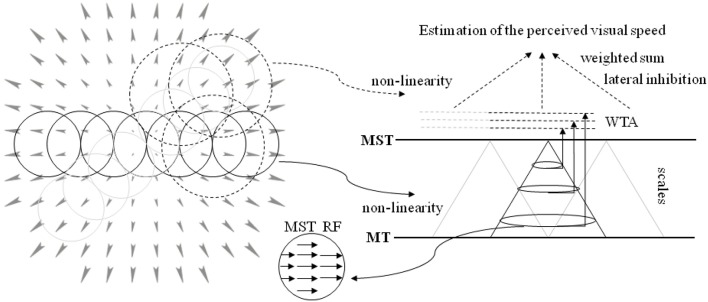
A sketch of the proposed model. **(Left)** The circular RFs of the model are superimposed on the visual stimulus (optical flow, expansion). The RFs tile all the visual field: the solid line circles denote the MST RFs, and the dotted circles denote the area, where the MST activity is processed. **(Right)** The neural architecture to estimate the speed of forward translation during self-motion (see text for details). The activity of MT visual area is processed by the MST RFs that perform an adaptive template matching (in the middle inset a template is shown) to detect variation of velocity. A multi-scale approach is adopted. The population of each MST scale is processed through a WTA, and such outputs are used in a weighted sum to estimate the forward translation motion, i.e., the perceived visual speed.

Here, we propose a novel neural model that processes the output of the aforementioned layers (see Solari et al., 2015; Chessa et al., 2016a,b for details) for the estimation of the perceived visual speed. The proposed computational neural model can be summarized as follows (see Figure 3 for a sketch of the proposed model):

- The population of MST cells at different scales performs an adaptive template matching (e.g., see the example of a MST RF in Figure 3) on the MT motion patterns that take into account a non-linearity to describe the space-variant resolution of retinas (Solari et al., 2012, 2014).
- An approach is adopted, in order to take into account both the evidence that MST RFs have different sizes and the fact that the visual signal contains information at different spatial scales.
- The activity of the MST cells is locally processed by a Winner-Take-All (WTA) approach: specifically, the WTA is locally applied on the sub-populations of each scale. Moreover, a compressive non-linearity is applied on the WTA outputs.
- In order to estimate the perception of speed of forward translation during self-motion, the most active scale is selected and its spatial neural activity is pooled through a weighted sum: in particular, we consider both positive and negative weights (i.e., there is an inhibition due to the activity in the periphery of the visual field).

#### 2.2.1. Modeled MST Area

The dorsal MST area is associated with the specialized function of encoding visual cues to self-motion: in particular, there are neurons that are selectively sensitive to specific components (i.e., elementary components of optical flow patterns, as expansion, contraction, rotation, and translation) of the optical flow that occurs during self-motion (Tanaka et al., 1989; Duffy and Wurtz, 1991; Pitzalis et al., 2013; Cottereau et al., 2017), but (Wall and Smith, 2008) identified also two other areas sensitive to egomotion in humans. Several biologically plausible models of the MST functionality have been proposed (Perrone and Stone, 1994; Grossberg et al., 1999; Yu et al., 2010; Mineault et al., 2012). Specifically, we consider the approach presented in Chessa et al. (2013) and extend it to model the experimental data we present in our current work.

**Cortical representation** We consider the representation of optical flow as provided by a bio-inspired model (Chessa et al., 2016b) and we model the space-variant resolution of retinas by using the blind spot approach, i.e., log-polar mapping (Solari et al., 2012).

**Figure 4:**
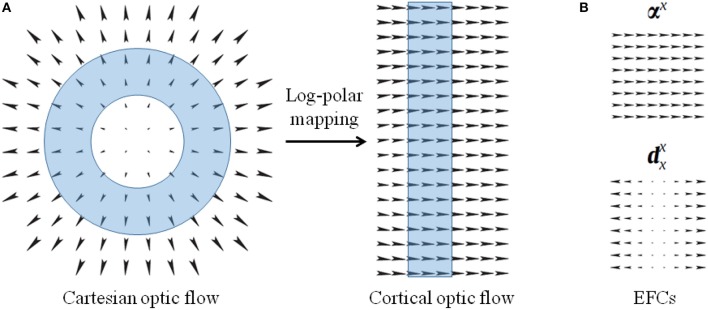
**(A)** Optical flows representing expansion in the Cartesian domain and the corresponding cortical optical flow. The log-polar mapping transforms Cartesian annular regions (cyan ring-shaped area) into cortical vertical stripes. **(B)** Example of elementary flow components representing cardinal deformations of the optical flow.

The log polar mapping modifies the Cartesian polar coordinates by applying a non-linearity on the radius ρ, as ξ = log_*a*_(ρ/ρ_0_), and a normalization on the angle coordinate θ (Schwartz, 1977; Traver and Pla, 2008; Solari et al., 2012, 2014). The transformation of a vector field from the Cartesian domain to the cortical domain can be expressed in terms of a general coordinates transformation (Chan Man Fong et al., 1997; Solari et al., 2014):

(1) $$\left[\begin{array}{c} v_{x}\\ v_{y} \end{array}\right]=\frac{1}{\rho_{0}a^{\xi }\ln(a)}\left[\begin{array}{cc} \cos\theta & \sin\theta \\ -\sin\theta & \cos\theta \end{array}\right]\left[\begin{array}{c} v_{xCart}\\ v_{yCart} \end{array}\right],$$

where *a* parameterizes the non-linearity of the mapping, and ρ_0_ is the radius of the central blind spot. *v*_*xCart*_ and *v*_*yCart*_ denote the components along *x* and *y* axes of the Cartesian optic flow, and the *v*_*x*_ and *v*_*y*_ components describe the transformed cortical optic flow. The scalar coefficient of Equation (1) represents the scale factor of the log-polar vector, and the matrix describes the rotation due to the mapping. It is worth noting that Cartesian annular regions of expansion optical flow that are centered around the fixation point, i.e., the fovea, are mapped into vertical stripes of horizontal optical flow in the uniform cortical representation (see Figure 4A). In Appendix some relevant optic flow patterns and their log-polar mappings are reported.

**Elementary flow components** The MST neurons are sensitive to elementary flow components (EFCs), such as expansion, shear, and rotation, or their combination with translation components (Koenderink, 1986; Orban et al., 1992). Since such EFCs can be described in terms of affine descriptions (Chessa et al., 2013), we describe the optical flow *v*(*x, y*) as linear deformations by a first-order Taylor decomposition, around each point: $v=\bar{v}+\bar{\text{T}}x$, where $\bar{\text{T}}$ is the tensor composed of the partial derivatives of the optical flow.

By describing the tensor through its dyadic components, the optical flow can be locally described through two-dimensional maps (***m*** : R^2^ ↦ R^2^) representing elementary flow components:

(2) $$\begin{array}{l} v=\alpha^{x}{\bar{v}}_{x}+\alpha^{y}{\bar{v}}_{y}+d_{x}^{x}{\frac{\partial v_{x}}{\partial x}|}_{x_{0}}+d_{y}^{x}{\frac{\partial v_{x}}{\partial y}|}_{x_{0}}\\ \;+d_{x}^{y}{\frac{\partial v_{y}}{\partial x}|}_{x_{0}}+d_{y}^{y}{\frac{\partial v_{y}}{\partial y}|}_{x_{0}}, \end{array}$$

where, the first two terms are pure translations and the other ones are cardinal deformations, basis of a linear deformation space: for instance, **α**^*x*^:(*x, y*) ↦ (1, 0) and $d_{x}^{x}:\left(x,y\right)\mapsto \left(x,0\right)$ (see Figure 4B).

We can model the sensitivity to such deformations through a population of MST cells whose response is obtained by an adaptive template matching on the cortical optical flow. From the responses of such a population we compute the first-order (affine) description of the cortical optical flow (Koenderink, 1986; Orban et al., 1992).

**Affine flow model and motion interpretation** The affine description of optical flow is related to the interpretation of visual motion (Chessa et al., 2013): specifically, the affine coefficients can be combined in order to compute quantities related to the relative motion between an observer and the scene, such as the estimation of the 3-D orientation of the surfaces, of the time to collision, of the focus of expansion, and of the translational speed that is of interest for the current work.

To clarify the relationships, we can consider the following affine description of the optical flow:

(3) $$\left[\text{​​}\begin{array}{l} v_{x}\\ v_{y} \end{array}\text{​​}\right]=\left[\text{​​}\begin{array}{c} c_{1}\\ c_{4} \end{array}\text{​​}\right]+\left[\text{​​}\begin{array}{cc} c_{2} & c_{3}\\ c_{5} & c_{6} \end{array}\text{​​}\right]\;\cdot \;\left[\text{​​}\begin{array}{c} x\\ y \end{array}\text{​​}\right].$$

The relative motion between an observer and the scene can be described as a rigid-body motion: a 3D point **X** = (*X, Y, Z*)^*T*^ has a motion given by $\overset{∙}{\text{X}}=-\left(\text{T}+\Omega \wedge \text{X}\right)$, where $\text{T}={\left(T_{X},T_{Y},T_{Z}\right)}^{T}$ denotes the translational velocity and $\Omega ={\left(\Omega_{X},\Omega_{Y},\Omega_{Z}\right)}^{T}$ the angular velocity (Longuet-Higgins and Prazdny, 1980). By considering a pinhole camera model with focal length *f*, we obtain the perspective projection of the motion:

(4) $$\begin{array}{l} \left[\text{​​}\begin{array}{l} v_{x}\\ v_{y} \end{array}\text{​​}\right]=f\left[\text{​​}\begin{array}{c} -T_{X}/Z-\Omega_{Y}\\ -T_{Y}/Z+\Omega_{X} \end{array}\text{​​}\right]+\left[\text{​​}\begin{array}{cc} T_{Z}/Z & \Omega_{Z}\\ -\Omega_{Z} & T_{Z}/Z \end{array}\text{​​}\right]\;\cdot \;\left[\text{​​}\begin{array}{c} x\\ y \end{array}\text{​​}\right]\\ \;+\frac{1}{f}\left[\text{​​}\begin{array}{c} xy\Omega_{X}-x^{2}\Omega_{Y}\\ y^{2}\Omega_{X}-xy\Omega_{Y} \end{array}\text{​​}\right]. \end{array}$$

By considering a smooth surface structure, specifically we locally approximate the surface through a planar surface, we can describe the affine coefficients in terms of the motion quantities of Equation (4) (Chessa et al., 2013). In particular, the affine coefficient *c*_2_ (in the condition of the experiment considered in this work) is proportional to *T*_*Z*_, i.e., the forward translation speed in an ego-motion scenario.

The coefficient *c*_2_ can be estimated through a template matching by using the map $d_{x}^{x}$ that describes the MST RFs of a population of cells. Thus, the output of such a template matching can be considered as the MST neural activity *E*(*p*), where *p* = (*x, y*) denotes the domain, i.e., the coordinate reference system.

#### 2.2.2. Modeled Perceived Visual Speed

To take into account the experimental data about the range of the RF size (Raiguel et al., 1997), we consider four scales (*s*) in the range 10−50°, thus the MST neural activity is described as *E*(*p, s*). Moreover, we implemented a multi-scale approach also to consider the fact that the visual signal contains information at different spatial scales.

With the aim of obtaining an estimate of perceived forward translation speed, the distributed neural MST activity *E*(*p, s*) is processed through a Winner-Take-All approach. Specifically, we locally apply a WTA on the neural sub-population of each scale: the WTA processes the MST activity on an area *W* of 70° with 75% overlap. Moreover, a compressive non-linearity β is applied on the WTA output:

(5) $$E_{WTA}(p,s)={\left(\underset{p\in W}{\max}\;*\;E(p,s)\right)}^{\beta },$$

where * denotes that the WTA is applied by using a moving window *W*.

To exploit the information gathered by the multi-scale approach, we model a WTA layer that selects the most active neural sub-population among the ones of the considered scales:

(6) $$E_{WTA}(p)=\underset{s}{\max}\;E(p,s).$$

We propose a spatial pooling of the MST activity to obtain a scalar value *Pz* as an estimate of the perceived forward translation speed. In particular, the activity in the visual periphery (area *W*_*p*_) has an inhibitory role with respect to the central area *W*_*c*_ (see section 2.2.3 also), if there is an activity in a small area *W*_*f*_ around the fovea:

(7) $$Pz=\sum_{p\in W_{c}}E_{WTA}(p)+g(\underset{p\in W_{f}}{E_{WTA}(p)})\sum_{p\in W_{p}}E_{WTA}(p),$$

where *g*(·) denotes a gating function that has a negative value when there is an activity in the area *W*_*f*_ around the fovea (otherwise it assumes a positive value). In the current implementation, we have *W*_*f*_ = 5°, *W*_*c*_ = 85° (i.e., a central 85° area) and *W*_*p*_ = 25° (i.e., peripheral 25°).

**Comparison with human data** The model estimate *Pz* of the perceived forward translation speed (see Equation 7) can be directly compared with the human estimates of the described experiment: **Figure 9** shows the model estimates *Pz* for the 13 visual stimulus conditions with respect to the corresponding human data. To provide a measure of the difference between human and model data (i.e., the simulation error), we compute the *Pz* for the *N* = 13 visual conditions and we evaluate the relative error *e*_*mh*_ as follows:

(8) $$e_{mh}=(1/N)\sum_{i=1}^{N}|HD_{i}-Pz_{i}|/HD_{i},$$

where |·| denotes the absolute value, *HD*_*i*_ denotes the average human perception of speed for the i-th condition and *Pz*_*i*_ the model estimate for the same condition. All simulations were performed using the Matlab software.

#### 2.2.3. Systematic Analysis of the Influence of Processing Stages on the Model Performance

In order to understand how the different processing stages affect the model performance in modeling human estimates, we selectively remove specific processing stages of the proposed neural model and analyze the resulting outputs with respect to human data.

**Figure 5:**
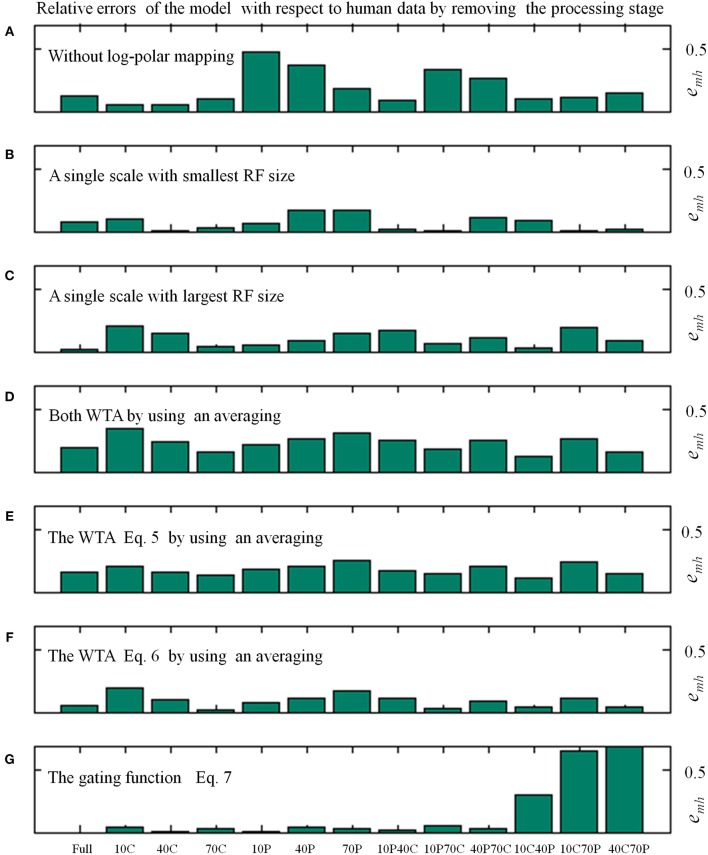
Relative errors (Equation 8) of the proposed model with respect to human data as a function of the 13 visual stimulus conditions by varying the processing stages, as in Table 1. In particular, the average relative errors by removing specific processing stages are as follows: about 4% (i.e., 0.036 by using Equation 8) for the full model; 19% without log-polar mapping **(A)**; 7 and 11% with a single scale, the smallest **(B)** and largest **(C)** RF size, respectively; 23% by changing both WTA with an averaging **(D)**; 18 and 9% by changing only one WTA **(E,F)**; 15% without gating function **(G)**.

Table 1 shows the average relative error *e*_*mh*_ (see Equation 8) of the model in replicating the human data by removing specific processing stages. In Figure 5 the distribution of the relative errors on the 13 stimulus conditions is shown, for the same model changes as in Table 1.

**Table 1:** Average relative errors (Equation 8) of the proposed model with their standard deviations as a function of the processing stages.

**Removal of the processing stage**	***e*_*mh*_ ± its std**
**Full model**	**0.036** ± **0.034**
Log-polar mapping	0.186 ± 0.135
A single scale with the smallest RF size	0.068 ± 0.059
A single scale with the largest RF size	0.107 ± 0.065
Both WTA, Equations (5) and (6) (by using an averaging)	0.229 ± 0.066
WTA, Equation (5) (by using an averaging)	0.179 ± 0.043
WTA, Equation (6) (by using an averaging)	0.090 ± 0.052
Gating function, Equation (7)	0.148 ± 0.251

The removal of the log-polar mapping affects the performances of the model in mimicking the human data: indeed, the average relative error is *e*_*mh*_ = 0.186 with respect the full model that has *e*_*mh*_ = 0.036 (see Table 1). By looking at Figure 5A we can see that the conditions 10P and 40P (also 10P70C and 40P70C) are hugely affected, indeed they are the areas between fovea and periphery, where the log-polar mapping mainly acts.

Conversely, to use a single scale instead of four scales has a smaller impact on the model performances and the effect on the different visual conditions is uniform, Figures 5B,C. By using a single scale with the smallest RF size (i.e., 10°) produces an average relative error *e*_*mh*_ = 0.068. The error is *e*_*mh*_ = 0.107 with the largest RF (i.e., 50°).

To change the WTA approach with an averaging affects hugely the model performances by causing an average relative error *e*_*mh*_ = 0.229, however the effect on the visual conditions is uniform (see Figures 5D–F). By removing the WTA that acts within each scale (Equation 5) has the most effect (*e*_*mh*_ = 0.179) with respect the WTA that acts among scales (Equation 6, *e*_*mh*_ = 0.090).

The removal of the gating function (see Equation 7) has a medium impact on the model performances, i.e., *e*_*mh*_ = 0.148. Nevertheless, it affects in an asymmetric way the relative errors on the visual conditions (see Figure 5G): the visual conditions 10C40P, 10C70P, and 40C70P are the most affected. For such conditions both the central and the peripheral part of the visual scene are visible: this suggests that might be present an (inhibitory) interaction between the foveal and peripheral areas.

## 3. Results

### 3.1. Influence on the FoV on Perceived Visual Speed With Dots (Optical Flow Only)

**Figure 6:**
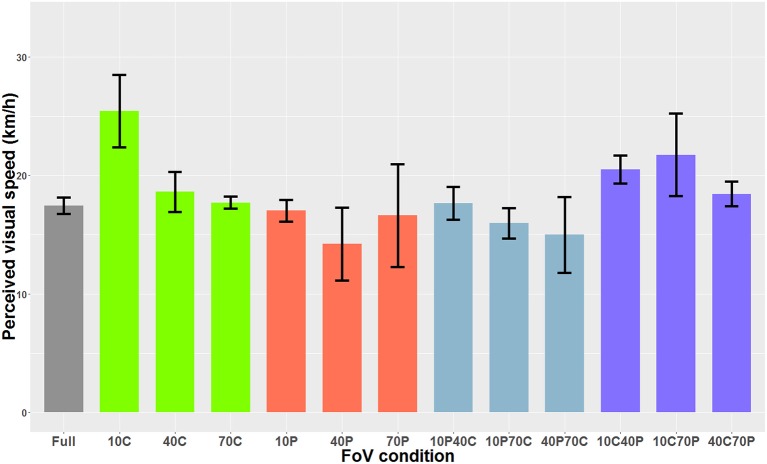
PSE mean values as a function of the FoV when the optical flow consisted of dots (i.e., optical flow only). The error bars represent the standard error of the mean.

When the optical flow consisted of dots, the Shapiro-Wilks test performed on the residuals indicated that data was not normally distributed. The Friedman rank sum test indicated a main effect of the FoV condition (i.e., the type of field of view) on perceived visual speed [χ^2^_(12)_ = 56.60, *p* < 0.001]. *Post-hoc* tests performed with the Friedman multiple comparisons function indicated that in the 10C condition, the PSE was significantly higher than in the 10P, 40P, 10P70C, and 40P70C conditions. In other words, the optical flow was perceived as significantly slower in the 10C condition than in the 10P, 40P, 10P70C, and 40P70C conditions. In addition, the optical flow was perceived as significantly slower (i.e., higher PSE) in the 10C40P than in the 40P and 10P70C condition. Finally, the optical flow was perceived as significantly slower in the 10C70P condition than in the 40P, 10P70C, and 40P70C conditions. Figure 6 shows the PSEs for all 13 FoV conditions.

### 3.2. Influence on the FoV on Perceived Visual Speed With 3D Spheres (Optical Flow + Expansion Cues)

**Figure 7:**
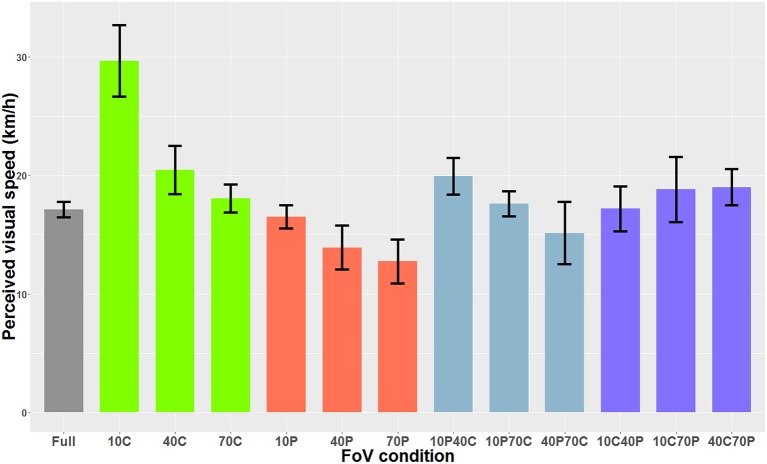
PSE mean values as a function of the FoV when the optical flow consisted of 3D sphere (i.e., optical flow + expansion cues). The error bars represent the standard error of the mean.

When the optical flow consisted of 3D spheres and included optical expansion cues, the Shapiro-Wilk analysis indicated that the residuals were normally distributed. The one-way ANOVA indicated a main effect of the FoV condition on perceived visual speed [*F*_(12, 84)_=47.37, *p* < 0.001]. Bonferroni-corrected paired-comparisons indicated that in the 10C condition, the optical flow was perceived as significantly slower (i.e., higher PSE) than in all other FoV conditions. Also, the optical flow was perceived as significantly faster in the 40P and 70P FoV conditions than in the 40C, 70C, 10C40P, 10C70P, 40C70P, and 10P40C conditions. Figure 7 shows the PSEs for all 13 FoV conditions.

### 3.3. Direct Comparison of Perceived Visual Speed With Dots and 3D Spheres

**Figure 8:**
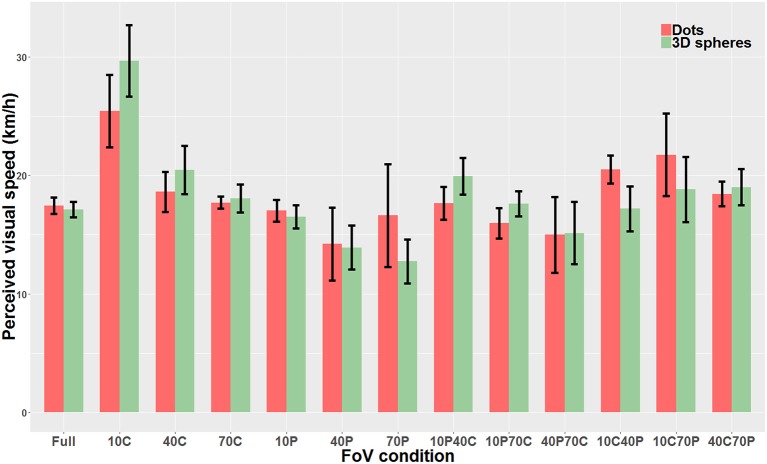
Direct comparison of PSE mean values measured with dots (red) and 3D spheres (green). The error bars represent the standard error of the mean.

We then compared “directly” the Dots condition with the 3D spheres condition. Because data was non-parametric and we had a repeated measures design, we used a linear mixed model. The analysis revealed that the type of visual stimulus used for the optical flow (i.e., dots vs. 3D spheres) did not have any effect on perceived speed (χ^2^_(1)_=0.0004, *p* = 0.98). On the other hand, there was a main effect of the type of FoV (χ^2^_(12)_=226.06, *p* < 0.0001) as well as a significant interaction between the two main factors (χ^2^_(12)_=58.12, *p* < 0.0001). Therefore, for each FoV condition, we directly compared the PSE measured with dots and the PSE measured with 3D spheres. These tests were performed using paired *t*-tests or Wilcoxon signed-rank test (when data was non-parametric). These tests were Bonferroni-corrected for multiple comparisons. None of the 13 tests indicated a significant difference between the PSE measured with dots and the PSE measured with 3D spheres. The only FoV condition for which the test was close to reaching significance (*p* = 0.063) was the 10C condition. Note that using a two-way repeated measures ANOVA instead of the linear mixed model gave the exact same pattern of result, namely no effect whatsoever of the type of stimulus (i.e., dots vs. 3D spheres) on perceived speed (*p* = 0.99), a main effect of the type of FoV (*p* < 0.001) and an interaction between the two main factors (*p* < 0.001). Figure 8 shows perceived speed for all FoV conditions and with the two types of visual stimuli.

### 3.4. A Computational Model of Motion Processing Accounts for the Patterns of Human Data

**Figure 9:**
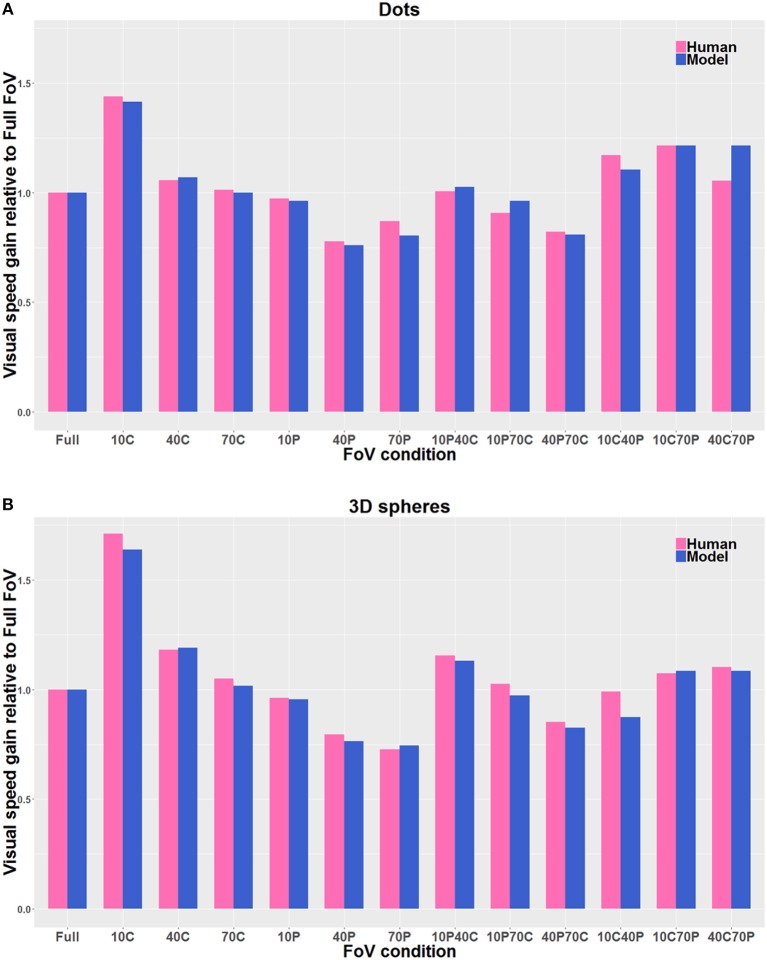
Direct comparison of the visual gains measured with the participants (Human) with the visual gains given by the model (Model), both when the visual stimulus consisted of Dots **(A)** and of 3D spheres **(B)**. The pink bars correspond to the human data, and the blue bars correspond to the model estimates. The gain values are directly derived from the PSE values, so that gain values smaller than 1 indicate an overestimation of visual speed (relative to Full FoV), and gain values larger than 1 indicate an underestimation of visual speed. The visual stimulus conditions are reported in the text. The average relative error is about 4% by using Equation 8.

Figure 9 shows the estimates of perceived visual speeds of the proposed model (i.e., *Pz*, see section 2.2 for details), assessed by using the same stimuli and procedure as the human observers. The underestimation and overestimation of speed exhibited by the model are very similar to the ones of human observers: in particular, the model is able to replicate the human behavior for 10C, 10C70P, 10P40C, 40C, and 40C70P, but 10C40P shows a larger error, though acceptable. In general, the proposed computational model shows a high level of agreement with the human data: the average relative error *e*_*mh*_ is about 4% (i.e., 0.04 by using Equation 8).

## 4. Discussion

Participants were presented with an optical flow constituted of limited-life-time random dots or 3D spheres moving in their direction along the antero-posterior axis. The size and portion of the moving FoV was systematically manipulated. For all FoV conditions, we did not observe any significant difference between the two types of visual stimuli, namely dots and 3D spheres. In other words, irrespective of the size and portion of the displayed FoV, visual speed perception was similar whether only optical flow information was available (i.e., Dots condition), or additional expansion/looming cues were present (i.e., 3D spheres conditions). On the other hand, both the size and portion of the moving visual field affected visual speed perception. In particular, patterns in which only the central part of the visual field was moving resulted in a larger underestimation of flow speed. Importantly, a bio-inspired computational model of the neural processing stages of the dorsal pathway allowed us to predict perceived speed based on the visible portion of the moving optical flow, and this with a 96 percent reliability.

Our results show that the size and the portion of visible FoV significantly affect perceived visual speed. In particular, as already described by Pretto and colleagues (Pretto et al., 2009), the wider peripheral-only conditions (namely 40P and 70P) resulted in an overestimation of the speed of the optical flow. However, and contrary to what was described by Pretto et al., this overestimation was significant only when the visual stimulus consisted of 3D spheres (i.e., when expansion cues were provided), and not when the visual stimulus consisted of dots. When only the central 10° of FoV were displayed (i.e., FoV condition 10C), we also found an effect on perceived visual speed, that was significantly underestimated as compared to most other FoV conditions, depending on the type of visual stimulus. With 3D spheres, the underestimation (in the 10C condition) was significant as compared to all other FoV conditions. With dots however, the underestimation was significant as compared to the FoV conditions in which a small portion of the central FoV was covered (i.e., 10P, 40P, 10P70C, 40P40C), but not as compared to the Full FoV condition. This result is at odds with the 2009 study by Pretto and colleagues who using dots, found that all FoVs smaller than 60° gave rise to a significant underestimation of visual speed as compared to the Full FoV condition. Overall, our results indicate that visual speed tend to be underestimated when only a small central portion of the FoV is visible. Several studies have highlighted the importance of peripheral vision for motion perception, with a direct influence on speed perception (Pretto et al., 2009, 2012), but also on navigation abilities (Czerwinski et al., 2002; Turano et al., 2005) and on vection, i.e., the sensation of self-motion that derives from a moving stimulus (Brandt et al., 1973; Berthoz et al., 1975; Mohler et al., 2005). In line with this, the underestimation of visual speed that we observed when the peripheral part of the FoV was occluded likely results from the fact that in this situation, only the low angular velocities of the visible central portion can be used for speed estimation, thereby “biasing” perception.

Importantly, using a biologically-inspired model, we were able to predict the influence of the size and portion of the moving visual field on speed perception. Specifically, by providing the appropriate parameters of the neural processing stages, our model allowed us to predict with 96% of reliability the perceived speed based on the visible portion of the moving optical flow. In the past, different computational models have been proposed to “explain/describe” the processes underlying human perception of visual speed, mainly by focusing on local computation of motion. Commonly, these models assumed that the perception of visual motion is optimal in one of two conditions: (i) in a deterministic framework with a regularization constraint induces the solution to bias toward zero motion (Yuille and Grzywacz, 1988; Stocker, 2001); (ii) in a probabilistic framework of Bayesian estimations, which a prior that favors slow velocities (Simoncelli, 1993; Weiss et al., 2002). Other studies have shown that it is possible to capture basic qualitative features of translational motion perception with an ideal Bayesian observer model based on Gaussian forms for likelihood and prior (Weiss et al., 2002). Because the previous model deviates from human perceptual data regarding trial-to-trial variability and the form of interaction between perceived speed and contrast, Stocker and Simoncelli (2005) proposed a refined probabilistic model that could account for trial-to-trial variabilities. These authors derived the prior distribution and the likelihood function of speed perception from a set of psychophysical measurements of speed discrimination and matching.

Nevertheless, in order to perceive motion patterns that are related to visual navigation, one should consider a hierarchical processing and a spatial integration of the local motion, as described by previous models. Indeed, several models take into account the MST functionality and its larger receptive fields (Perrone and Stone, 1994; Grossberg et al., 1999; Yu et al., 2010; Mineault et al., 2012). In their seminal work, Perrone and Stone (1994) introduced a template-based model of self-motion that showed similar responses properties to MST neurons. In Grossberg et al. (1999), the model considers also log-polar mapping, though by using a formulation that does not allow a signal processing description as in our model. In Yu et al. (2010) and Mineault et al. (2012), the authors analyzed different types of neural combinations of local motion processing in order to account for the observed stimulus selectivity of MST neurons. It is worth noting that our model allows the prediction of perceived visual speed considering also the size and portion of the visual field. To obtain such a result, we have introduced several neural mechanisms by combining them in a novel computational model. In particular, we model a population of MST cells that perform an adaptive template matching by considering the spatial non-linearity produced by the log-polar mapping and multi-scale layers. Such a template matching allows a decomposition of motion patterns into an affine description that can be directly related to forward speed of the observer: the results show that the model estimates are similar to the perceived visual speed of human observers (i.e., the average relative error is about 4%).

Though there were some slight differences, the two types of visual stimuli, namely dots and 3D spheres, resulted in similar patterns of perceived visual speed. Specifically, providing expansion cues (3D spheres condition) in addition to the optical flow information did not alter perceived visual speed, and no significant difference could be observed between the Dots and the 3D spheres conditions. The only FoV condition for which a difference coming close to significance could be observed was the 10C condition, i.e., the FoV condition in which only the central 10° of FoV were visible. Note that this “tendency” could simply be due to the fact that in the 3D spheres condition, optical flow information might have been reduced because of the rapid expansion of the sprites which tended to cover the “small” visible area. This absence of significant difference between the Dots and the 3D spheres suggests that to estimate visual speed, at least in the conditions of the experiment, i.e., with simple visual stimuli, the optical flow provides sufficient motion information, and expansion cues do not provide much additional “benefit.”

## Data Availability Statement

The datasets generated for this study are available on request to the corresponding author.

## Ethics Statement

The studies involving human participants were reviewed and approved by Max Planck Institute for Biological Cybernetics and University of Tuebingen. The patients/participants provided their written informed consent to participate in this study.

## Author Contributions

PP, J-PB, MCh, and FS conceived and designed the study. J-PB and PP collected the human participants data. MCa, PP, and J-PB analyzed the data. MCh and FS developed the neural computational model. All authors contributed to the drafting of the manuscript.

### Conflict of Interest

PP was employed by Virtual Vehicle Research Center, Graz, Austria. The remaining authors declare that the research was conducted in the absence of any commercial or financial relationships that could be construed as a potential conflict of interest.

## Figures and Tables

**Figure A1:**
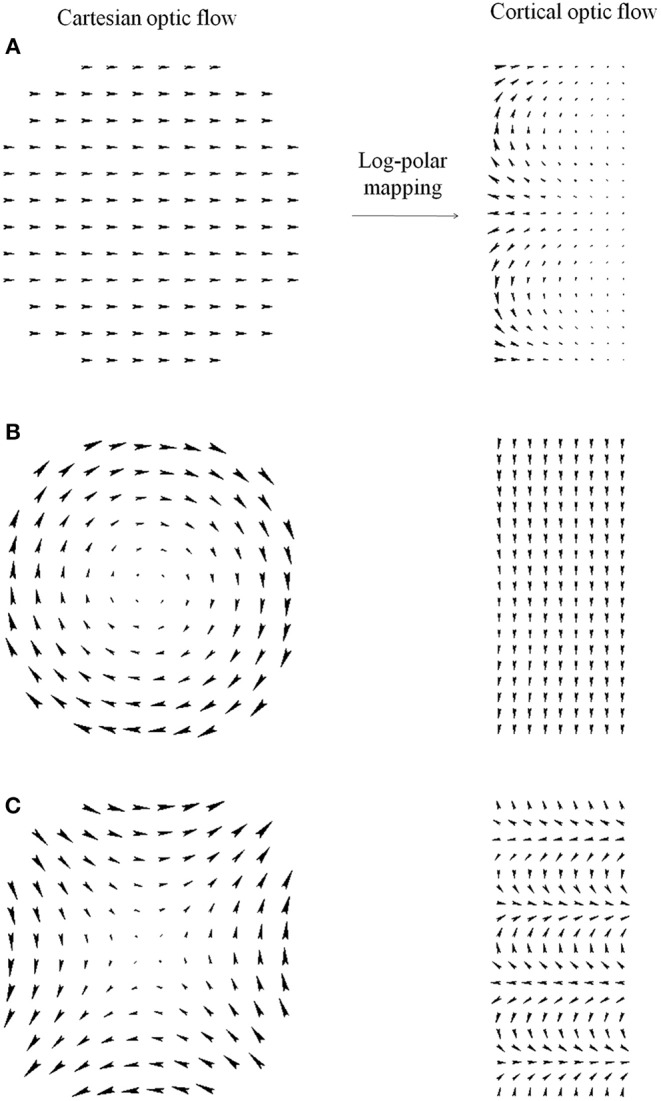
Optic flow patterns in the Cartesian and cortical domains: **(A)** constant, **(B)** rotation, and **(C)** shear optic flow pattern.

## Appendix

Figure A1 shows how the Cartesian optic flow patterns are transformed into the cortical domain. In particular, Figure A1A shows that constant optic flows in the Cartesian domain map to non-linear flows in cortical domain. Whereas, expansion (see Figure 4) and rotation (Figure A1B) flow patterns are mapped to constant flows along the horizontal and the vertical cortical axes, respectively. In general, a Cartesian optic flow is warped in the cortical domain, e.g., in Figure A1C the transformation of a Cartesian shear pattern is shown.
